# Fused Filament Fabrication of Alumina/Polymer Filaments for Obtaining Ceramic Parts after Debinding and Sintering Processes

**DOI:** 10.3390/ma15207399

**Published:** 2022-10-21

**Authors:** Claudio Tosto, Mario Bragaglia, Francesca Nanni, Giuseppe Recca, Gianluca Cicala

**Affiliations:** 1 Department of Civil Engineering and Architecture, University of Catania, Viale Andrea Doria 6, 95125 Catania, Italy; 2 Department of Enterprise Engineering “Mario Lucertini”, University of Rome “Tor Vergata”, INSTM RU Roma—Tor Vergata, Via del Politecnico 1, 00133 Rome, Italy; 3 Institute for Polymers, Composites and Biomaterials, IPCB-CNR, Via Gaifami 18, 95126 Catania, Italy

**Keywords:** ceramic, 3D printing, fused filament fabrication, mechanical properties

## Abstract

In this paper, a hybrid commercially available alumina/polymer filament was 3D printed and thermally treated (debinding and sintering) to obtain ceramic parts. Microscopic and spectroscopic analysis was used to thoroughly characterize the green and sintered parts in terms of their mesostructured, as well as their flexural properties. The sintered samples show an α alumina crystalline phase with a mean density of 3.80 g/cm^3^, a tensile strength of 232.6 ± 12.3 MPa, and a Vickers hardness of 21 ± 0.7 GPa. The mean thermal conductivity value at room temperature was equal to 21.52 ± 0.02 W/(mK). The values obtained through FFF production are lower than those obtained by conventional processes as the 3D-printed samples exhibited imperfect interlayer bonding and voids similar to those found in the structures of polymeric FFFs. Nonetheless, the highly filled ceramic filament is suitable for use in affordable and easy-to-operate FFF machines, as shown by the cost analysis of a real printed and sintered FFF part.

## 1. Introduction

Due to their composition and structural characteristics, as well as their highly controlled processing conditions, technical ceramics, also referred to as advanced ceramics, exhibit high performance in various applications [1].

Technical ceramics commonly used in a variety of industrial applications include alumina (Al_2_O_3_) [2,3], zirconia [4] (ZrO_2_, primarily yttria-stabilized tetragonal zirconia polycrystal, Y-TZP), and their composites, zirconia-toughened alumina (ZTA) and alumina-toughened zirconia (ATZ) [5].

The most extensively researched and used technical ceramic is probably alumina. It can be used in various fields thanks to its possession of characteristics such as bio-inertness and chemical resistivity, combined with good mechanical strength. Alumina components are used in electrical and electronic applications [6,7], membrane [8] and filtration products [9], and wear-resistant products like sandblasting nozzles, seal faces, bearings, and piston plungers [10].

Hence, ceramics manufacturing is a growing industry. According to a recent review by Holero et al. [11], a total of 1110 patents on ceramics were published between 2008 and 2018 [12,13], with the majority focusing on the development of material formulations for additive manufacturing (AM) technologies, followed by patents on process innovations, new applications for ceramic parts made by additive manufacturing, and equipment advancements. In fact, due to the use of diamond cutting and grinding tools, which are estimated to represent more than 70% of the total manufacturing costs, complex shapes can only be produced through time-consuming machining, which also entails significant increases in waste and production costs [12,13]. It can be challenging to machine-sinter components without introducing flaws and surface microcracks, because of their high hardness and brittleness. Therefore, the majority of polycrystalline advanced ceramics are made using the powder processing route, whether they are made from dry powders or powder slurries.

A well-known method for creating even tiny objects from ceramic materials is powder injection moulding (PIM), which, in the case of ceramic, is commonly known as ceramic injection moulding (CIM) [14,15,16,17]. CIM is like injection moulding for polymers, but different in the fact that the granular pellets are made of polymer binders and ceramic powders. Particularly for large quantities, this procedure is very cost-effective [15]. Due to the expensive moulds, tools and extended production times, this technique is unaffordable for small lot sizes or customised parts like prototypes [17]. Rapid tooling, a technique that uses common 3D printing techniques to create tools from plastic materials, is one way to obtain small quantities [17,18,19,20,21,22].

By providing an alternative to traditional formative processes, AM enables the production of geometrically complex near-net-shape 3D ceramic parts without the need for costly tooling. However, because additively manufactured ceramic parts have poor resolution, surface quality, mechanical properties, and scalability when compared to traditional ceramic manufacturing processes, the adoption of AM technologies in the ceramic industry has been much slower than in the polymer and metal industries.

Ceramic materials can already be printed in three dimensions. The common procedures, such as stereo lithography (SLA), selective laser sintering (SLS), laminated object manufacturing (LOM), three-dimensional printing (3DP), direct ceramic ink-jet printing, and fused filament fabrication (FFF), are outlined by Tay et al., Travitzky et al., and Gonzalez-Gutierrez et al. [23,24,25].

Fused filament fabrication (FFF) can create a wide range of thermoplastic or composite objects with intricate geometries. The relationship between the process parameters, the finished part’s quality, and the part’s mechanical performance is crucial to investigate [26,27]. FFF printing of ceramics has demonstrated great promise as a cost-effective substitute for ceramic production [28,29], especially for businesses currently using PIM, which already have the tools and expertise to complete the following steps of binder removal and sintering to produce solid parts with intricate geometries [30,31]. Material extrusion AM of highly filled polymers (MEAM-HP), according to Gutierrez et al. [31], is intended to be used for the production of small quantities (<1000 parts per year, such as prototypes or custom-made parts) or parts with geometries that cannot be achieved by filling the cavity of a mould. As a result, the authors think that MEAM-HP will soon fill an industrial void, making it worthwhile to research and develop the equipment and feedstock materials used in this AM process.

Compared to other AM techniques, FFF printing of ceramics has several advantages. AM techniques that require the use of photopolymer resins (SLA, LCD—Liquid Crystal Display, and LCM—Lithography-based Ceramic Manufacturing) have the following disadvantages: liquid monomers can be harmful, material creeping can occur after curing, lengthy post-processing is needed, support structures might be needed, and the equipment is expensive. Among the advantages of FFF printing are the facts that the equipment is inexpensive, there are a great variety of materials, it is easy to use, small to large building spaces are feasible, it is not limited by the presence of the vat that contains the resin, and that there is a possibility of multi-material fabrication.

At the same time, as noted by Tymrak et al. [32], when the same filaments are printed on different FFF printers, additional variability is introduced due to the fact that each printer differs from each other with regard to mechanical design, including frame, stepper motors, and extruder head. Therefore, it is always better to expand the material testing on different printers to ascertain the average properties that can be achieved.

Depending on the ceramic material, solids loading can range from 45 to 65 vol%. The base polymer (e.g., polypropylene, polyethylene, etc.) is combined with an elastomer, a tackifier, and a plasticizer to form the binder system of an FFF feedstock. These components give the filament strength, elasticity, flexibility, and plasticity, while wax is also included to reduce viscosity [33]. Al_2_O_3_, SiO_2_, Si_3_N_4_, and PZT have all been used to fabricate 3D parts using FFF [32,33,34,35,36,37,38]. After the component is formed (called “green” parts), a controlled heating process called debinding is used to remove the organic binder, leaving behind a “brown” body that is then sintered by heating it up in a furnace to a temperature above melting point. This causes a physicochemical transformation that gives the component its final material properties and microstructure.

Similar to PIM/CIM, MEAM-HP can also be used to debind shaped parts, but the majority of results reported in the literature concern thermal and solvent debinding. Sintering is also carried out in the same manner as in other powder technologies, like PIM. To optimize the final shape and functionality of components generated by MEAM-HP, debound and sintered, it is crucial to note that there will be a large anisotropic shrinkage comparable to the one seen in PIM. Since layer height, temperatures, deposition direction, and infill grade, among other factors, will affect the characteristics and shape of the finished ceramic parts, the building job must be optimized by adjusting the processes parameters and the building strategy. When the entire process is optimized, MEAM-HP produces objects with acceptable properties when compared to the results of other shaping technologies [31].

To enable AM of monolithic ceramics, researchers are now increasingly concentrating their efforts. Developing and perfecting AM techniques and ceramic feedstocks that are specifically made to produce monolithic nearly net-shape technical ceramic parts is still a challenge. The production of ceramic parts with controlled microstructures and without limitations in terms of part dimensions and geometrical complexity should be optimized further to compete with plastic AM in terms of technological maturity.

Recently, some research groups have been concentrating on the preparation of composite ceramic filaments, studying their processing using FFF-based printers [19,30,31,33,34,35,36,37,38,39,40,41,42,43,44,45]. However, only a very limited number of papers have focused on developing a full understanding of their mechanical properties [45,46,47]. With respect to the investigation of thermal properties (i.e., thermal diffusivity, thermal conductivity), in the literature there is a great lack of works, except for that of Schwentenwein et al. [48], on samples printed using the LCM process.

The need to investigate thermal properties is immediately evident when observing applications in environments subjected to high temperatures [49], such as customized crucibles, heating cartridges, Venturi burners, susceptors for microwave sintering, and on-demand tools for welding shields.

Anisotropic behaviour in printed parts obtained using additive manufacturing is well known. Therefore, to successfully achieve commercial exploitation, ceramic AM techniques must also be characterized in terms of their anisotropic behaviour. The aim of this study is the preparation of lightweight alumina ceramics by means of FFF printing. The bulk density before and after the debinding and sintering (D&S) processes was related to flexural strength, as well as morphology, shrinkage evaluation, and thermal conductivity properties. Finally, the costs of FFF printing of ceramic parts were compared to those of other AM processes (LCM, LCD, and DLP).

## 2. Materials and Methods

### 2.1. Al_2_O_3_ Filament Characterization and FFF 3D Printing of Samples

**Figure 1:**
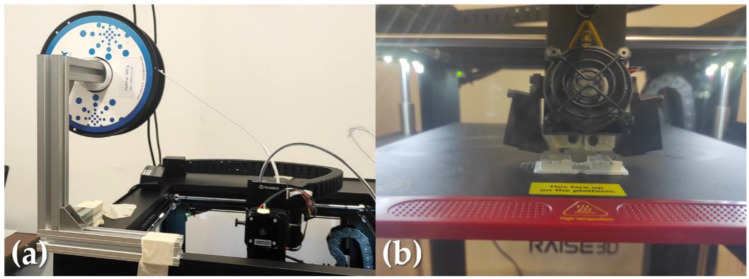
FFF printer: (**a**) direct feeding system, (**b**) building area.

**Figure 2:**
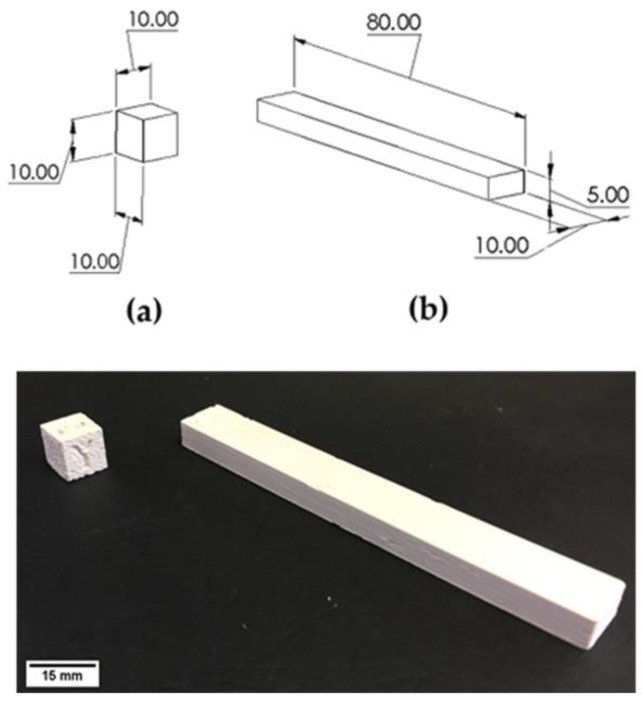
Sample sketch with nominal dimensions in mm (**top**) and 3D-printed samples (**bottom**): (**a**) cubes; (**b**) flexural samples.

The FFF feedstock, purchased from Nanoe (Ballainvilliers, France), was a filament filled with 52 vol% of Alumina powder [50]. All the samples (Figure 1 and Figure 2) were fabricated by FFF printing Raise3D Pro2 Plus (Irvine, CA, USA), with a direct feeding system and an arranged location of the spool to avoid breaking and failures during the print-head movements. The configuration of the spool system was changed in order to minimize failures due of the brittleness of the filament (Figure 1a). The printing settings used in the slicing software IdeaMaker by Raise3D for manufacturing the green parts are reported in Table 1. The values used for similar materials extruded by FFF are also reported.

As reported in Table 1, the printing temperature, except for the feedstock studied by Orlovskà et al. [47], confirms what other researchers have found. One of the most important parameters is the setting of the retraction, as also reported by Truxovà et al. [51], to limit the movement of brittle filament, and thus the possibility of breaks. Additionally, as described by the same authors, the feed mechanism’s pressure spring needed to be changed. The original spring harmed the filament, leading to issues during printing. A new spring with less pressure force resulted in less damage being caused to the filament, and the material was continuously fed without interruption.

**Table 1:** Printing setting adopted in the current study in comparison with similar FFF feedstock.

Infill Pattern	Infill Density	Walls	Overlap	Flow Ratio	Layer Thickness	Nozzle Diameter	Filament Diameter	Printing Speed	Platform Temperature	Printing Temperature	Particle Size	Solid Load	Binder	
*-*	*%*	*-*	*%*	*%*	*mm*	*mm*	*mm*	*mm/s*	*°C*	*°C*	*μm*	*vol%*	**-**	*Unit/References*
rectilinear	100	2	-	105	0.2	0.6	1.75	20	50	150	<1.0	52 (83 wt%)	**Polyolefine-based**	*Current study*
-	100	-	-	-	0.1	0.25		10	60	<170	0.1	50	**PEG/PVB**	*N*ö*tzel* et al., *2020* [52]
-	-	-	-	-	0.3	0.6		10	-	130–170	0.5	50	**EVA + stearic acid**	*Gorjan* et al. [53]
rectilinear	65	-	55	105	0.1, 0.2, 0.3	0.4		10–15	60	240	0.4–0.7	50	**wax/PE**	*Orlovskà* et al. [47]
-	100	-	-	-	0.1	0.25	2.85	2.5–10	65	135–180	0.1	45–55	**wax/PE + stearic acid**	*N*ö*tzel* et al., *2019* [54]
-	100	-	-	-	0.8	0.15	3	5	-	150–170	0.1	50 (86 wt%)	**wax/PE + stearic acid**	*N*ö*etzel* et al., *2018* [19]
rectilinear	100, 90, 80	2	40%	-	0.2	-	1.75	30	25	115–190 (150 opt)	<1.0	52 (83 wt%)	**Polyolefine-based**	*Truxová* et al. [51]
triangles, grid, honeycomb, rectilinear	5–100	≥2	-	-	0.1–0.3	0.4–1	1.75, 2.85	20–35	40	110–120	<1.0	52 (83 wt%)	**Polyolefin-based**	*Nanoe* [50]

The nominal dimensions of the samples, reported in Figure 2, are those desired after the D&S processes. All the samples were printed in consideration of the shrinkage occurring during the D&S processes. The formula used to calculate the oversize factors (*OFS*) was as follows:

(1) $$OFS=\frac{1}{1-S_{h}}$$

where *S_h_* is the shrinkage calculated from Equation (2):

(2) $$S_{h}=1-\frac{L_{s}}{L_{g}}$$

where *L_g_* and *L_s_* are the lengths of the green and sintered parts, respectively. In this step, the shrinkage factor (*S_h_*) was assumed to be equal to 17% and 21% in the plane and in the perpendicular (z) direction, respectively. Similar values have been reported in the literature for polyolefin-based filaments [30,31,40,41,47,50,51]. The *OFS* factors will be equal to 1.20 (OFS_xy_) and 1.26 (OFS_z_) in the plane and in the perpendicular direction, respectively.

After printing, all the samples were measured and weighed, in order to be able to control the densities and actual shrinkages, which will be discussed in the Results section.

The density measurements of the printed samples were performed according to the ASTM D79 buoyancy method, using Equation (3):

(3) $$\rho =\frac{w_{air}}{w_{air}-w_{liq}}\left(\rho_{L}-\rho_{0}\right)+\rho_{0}$$

where *ρ* is the density of the sample, *w_air_* is the weight of the sample in air, *w_liq_* is the weight of the sample in the auxiliary liquid, *ρ_L_* is the density of the auxiliary liquid, and *ρ*_0_ is the density of air. The percentage relative density was determined by calculating the ratio between the sample’s density *ρ* and the reference density of alumina (*ρ_alumina_ =* 3.95 g/cm^3^).

The sample’s porosity, *p*, was evaluated according to Equation (4):

(4) $$p=\left(1-\frac{\rho }{\rho_{alumina}}\right)\times 100$$

A high-vacuum emission scanning electron microscope (SEM EVO by Zeiss, Cambridge, UK) was used to examine the filament and 3D-printed samples cross-section. All the samples were gold sputtered to a thickness of 20 nm using an Emitech K-550 sputter coater (Ashford Kent, UK). 300× of magnification and a 15 kV accelerating voltage were used to collect the micrographs. 3D-printed flexural samples were tested by using a universal machine 5985 universal testing machine (Instron, Milan, Italy) equipped with a load cell of 10 kN and the tool described for the standard of flexural test (ASTM D790).

### 2.2. Debinding and Sintering of 3D-Printed Samples

The solvent debinding of 3D-printed samples was performed in acetone (ACS reagent, ≥99.5% Sigma Aldrich, St. Louis, MO, USA) at a temperature of 40 °C for 6 h under magnetic stirring.

Thermal debinding was performed according to filament processing guidelines [50] in a tubular furnace (Zsinter Nanone, France) in air atmosphere, in the temperature range 20–510 °C with different heating rate as reported in Table 2.

Sintering was performed at 1550 °C for 120 min, with a heating rate of 50 °C/h in air atmosphere. A rate of 100 °C/h from 1550 to 150 °C has been set for the cooling cycle.

### 2.3. Characterization of 3D-Printed Sintered Al_2_O_3_ Samples

Density measurements were carried out, according to ASTM D79, by means of buoyancy method-based pycnometer (Sartorius, Göttingen, Germany) allowing for the density determination applying Archimedes’ Principle. Mean density values were determined by performing three measurements on each specimen. The phase analysis of sintered 3D-printed samples was assessed by means of X-ray diffraction (XRD, Philips X’Pert 1710) in the 2θ range 10–90° in the following conditions: Cu Kα radiation (*λ* = 1.5408 Å), 40 kV and 40 mA, step size = 0.020°, time per step = 2 s. XRD measurements make it possible to evaluate the average crystallite size τ.

The *τ* value was obtained by applying Scherrer’s Equation (Equation (5)):

(5) $$\tau =\frac{K\lambda }{\beta \cdot cos\theta }$$

where *K* is the shape factor, taken to be 0.89, *λ* is the X-ray wavelength, *β* is the full width at half maximum (FWHM) of the most intense peak expressed in radians, and *θ* is the Bragg angle.

For the microstructure and morphological analysis, the 3D-printed sintered samples were sectioned using a precision diamond saw (Buehler IsoMet^TM^ 4000, Buehler, Lake Bluff, IL, USA); samples were cold-mounted in epoxy resin (Epoxy Infusion, curing time of 24 h at 25 °C) and mechanically polished with silicon carbide (SiC) papers and diamond suspension of up to 3 μm. After polishing, the samples were washed in ethanol ultrasonic bath. The surface morphology was imaged using electron microscopy (SEM EVO Zeiss) and EDS (Oxford EDS Inca X-sight, Oxford Instruments). ImageJ software was used to perform image analysis. The mechanical properties of sintered Al_2_O_3_ 3D-printed samples were evaluated by performing a three-point bending test according to ASTM D790 using a universal machine (Instron 5985, Milan, Italy), coupled with a 50 kN load cell. Tests were conducted on five samples, with the crosshead speed set at 1 mm/min with a span distance of 40 mm. The Vickers micro-hardness test (Future Tech FM-700, Future Tech Corp., Kawasaki, Japan) according to ASTM Standard E92 was carried out on five samples by applying a load of 500 g for 30 s along the cross-section of mounted samples, and ten measurements were performed on each sample.

The thermal diffusivity tests were performed on an LFA 467 HT HyperFlash machine (NETZSCH-Gertebau GmbH, Selb, Germany), in accordance with ASTM E1461, on five square samples with sides of 12.7 mm and a thickness of 2 mm, covered by graphite. Thermal diffusivity experiments were carried out at temperatures ranging from room temperature to 350 °C, with measurements taking place under isothermal conditions after every gap of 50 °C.

Equation (6) was used to calculate thermal conductivity:

(6) $$k=\alpha \cdot \rho \cdot C_{p}$$

where *α* is the thermal diffusivity (m^2^ s^−1^), *k* is the thermal conductivity (W m^−1^ K^−1^), *ρ* is the density (kg m^−3^), and *C_p_* is the specific heat capacity (J kg^−1^K^−1^), calculated by Differential Scanning Calorimetry (DSC) analysis, as reported in a previous work [55]. To calculate the thermal diffusivity, LFA Proteus analysis software was used with Equation (7) to process the time and temperature data collected during the test under adiabatic conditions:

(7) $$\alpha =0.1388\frac{s^{2}}{t_{0.5}}$$

where *s* is the thickness of the sample and *t*_0.5_ is the time needed to increase the temperature by 50%.

According to the conventional flash method [56], the pulse energy is completely absorbed on the specimen’s front face, and then flows through its thickness as a thermal wave before reaching the specimen’s opposite face. However, the pulse energy absorption is no longer limited to the front face, but rather extends over a thin layer into the specimen thickness, in materials that are slightly porous or have a rough surface. The absorption layer can be considered to be the material’s mean free path for photons. The consequence is the exponential decay of the specimen’s initial temperature distribution. The porous materials model takes into account the penetration effect and the subsequent decaying temperature distribution. Since the sample cannot be translucent in the visible and near-IR wavelength ranges, all the samples were coated with graphite before testing in order to minimise light reflection mistakes and optimise absorption and emission potential.

## 3. Results and Discussion

### 3.1. Filament and Green Part Characterization

**Figure 3:**
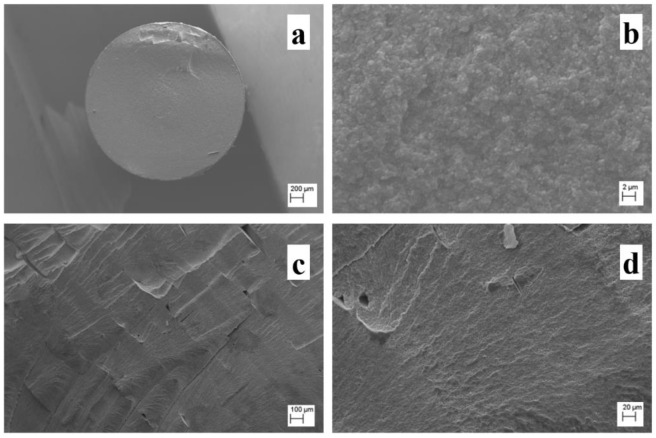
SEM images of: filament at magnifications of (**a**) 50× and (**b**) 5k×; green parts at magnifications of 100× (**c**), and 500× (**d**).

The filament cross-section was analysed by SEM (Figure 3a,b). From the scans, the Al_2_O_3_ particles appear to be homogeneously dispersed in the binder matrix, and it is possible to appreciate the particle size of the ceramic filler, analysed using the software ImageJ, and determined to be equal to 0.91 ± 0.08 μm. These values are the same as those reported by Nanoe [50] and Truxovà et al. [51]. They are greater than other filaments produced with similar wax-based binder matrix [28,47,48,49,52,53,54,57,58,59,60,61], and this can affect the printing quality, and therefore the final mechanical properties; as a result, it is very important to monitor the grain size after the sintering stage [60]. The external polymer coating revealed some highly filled filaments, and its use made it possible to consistently improve the flexural properties of the filament during printing [35]. For this reason, the filament studied in this paper is not suitable for Bowden-feeding systems, being better suited direct machines with modified positions for the spool, above the extrusion head.

After the printing, the green parts were observed via SEM, s shown in Figure 3c,d. The cross-section appears to be bulk in the centre, which indicates good bonding between the infill layers, but is porous in proximity to the wall lines.

### 3.2. Phase Analysis, Morphology, Density and Shrinkage of Sintered Samples

**Figure 4:**
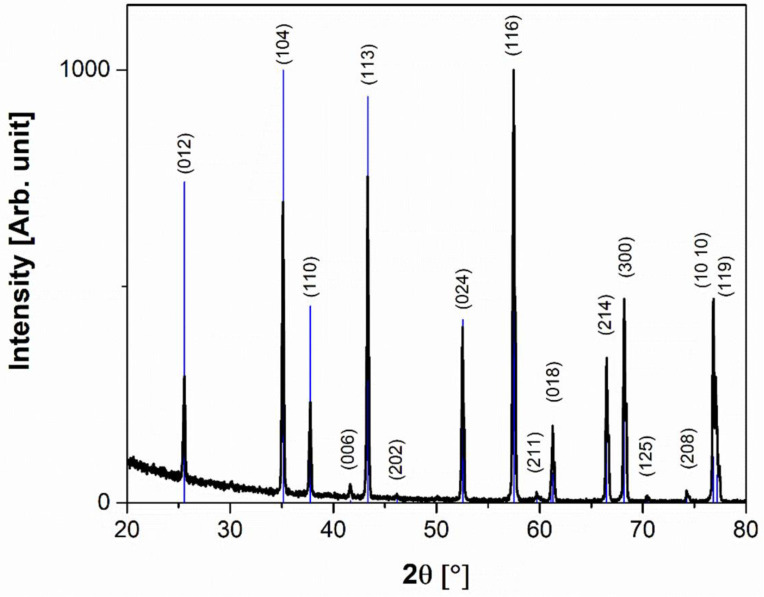
XRD pattern of 3D-printed sintered Al_2_O_3._

Figure 4 shows the XRD pattern of the sintered alumina sample. The reflections observed on the diffractogram highlight the presence of α alumina crystalline phase (JCPDS 88-0826). In the Al_2_O_3_ structure, the aluminium atom is octahedrally coordinated with oxygen atoms. The alumina structure can be seen as layers of hexagonal close-packed oxygen atoms, with aluminium atoms occupying two-thirds of the octahedrally coordinated holes between the oxygen atoms. Hence the atomic positions are 12 aluminium atoms and 18 oxygen atoms, respectively [62].

Alumina can exist in polymorphic phases (e.g., α, β, γ, δ, η), but most of them are metastable phases that are involved in transition sequences as a function of temperature, ending in the transformation to the stable α phase at a temperature of about 1050 °C [63]. In our case, the sintering temperature of 1550 °C fully justifies the presence of α phase, as no other peaks ascribable to other crystalline phases were detected.

The intensity ratios of the detected peaks are different from the randomly oriented ones in the reference pattern. This result suggests that a preferential orientation along the crystallographic direction takes place. This could be related to the FFF process, as the molten material is extruded through the nozzle and flattened on the plate to form the printed layer, and this may induce some degree of filler orientation that is retained after the sintering. The value of coherently scattering domain size calculated by applying Scherrer’s formula is 48 nm.

**Figure 5:**
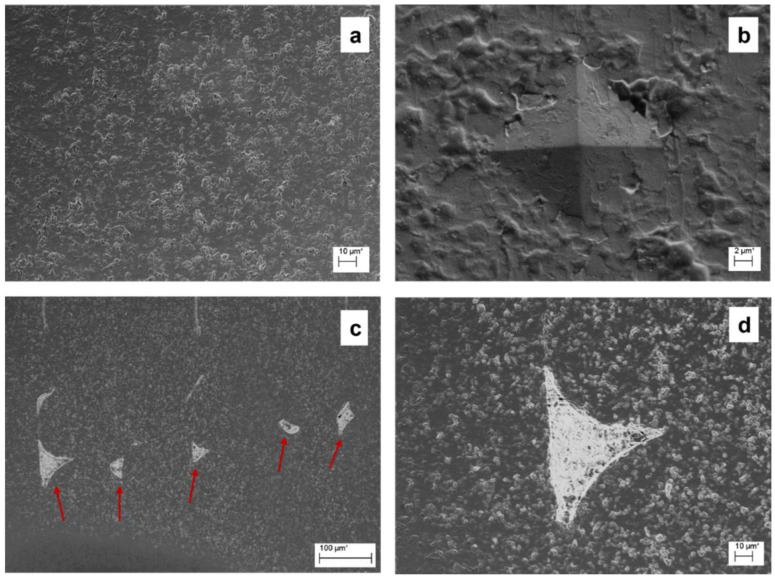
Morphology of sintered 3D-printed alumina sample observed by SEM. (**a**) Low magnification, (**b**) detail of Vickers hardness imprint, (**c**) porosity (arrows) as a result of the FFF process, (**d**) detail of porosity.

The mean value of the measured density of sintered 3D-printed samples was 3.80 ± 0.02 g/cm^3^. This is a good result, considering that the density value is very slightly lower than the bulk density of alumina (3.94–3.95 g/cm^3^), and this difference can be ascribed to the degree of porosity found in the 3D-printed samples, as highlighted by the SEM analysis presented in Figure 5c. In fact, some degree of porosity in specimens manufactured using FFF is an intrinsic drawback, especially when working with highly filled composite filaments. During FFF printing, the adhesion and the cohesion between new and previously deposited layers is dependent on the diffusion of polymer chains across the interface, and is related to the residual thermal energy in the material after deposition. These phenomena are directly dependent on viscous flow and are hence greatly influenced by viscosity, temperature, surface tension, road geometry, and thermal mismatch between deposited materials [64,65].

The higher the viscosity of the material, the higher the probability of obtaining some degree of porosity in the 3D-printed part [65,66]. It has also been proved that the degree of porosity is not homogeneous along the building direction (Z) of the sample, and therefore it is influenced by the height of the 3D-printed part [67]. Nevertheless, the density values were confirmed by the morphological characterization on the basis of SEM of the sintered samples. In fact, from Figure 5a, it is possible to observe that appropriate parameter settings for D&S processes (Table 2) result in to a cross-section characterized by very little void content. The voids detected, as shown in Figure 5c,d, are related to the FFF process, as confirmed by their triangular shape. In fact, the shape and type of porosity can provide information on the source determining it. It has been proved that if a pore is spherical, it is related to thermal treatment (debinding and sintering), on the other hand, if it is non-spherical, as in our case, the porosity is process induced (FFF printing) [40,68]. The sintered samples were measured after sintering and the dimensions in comparison to those recorded for the green samples are reported in Table 3.

It can be observed from Table 3 that the mean shrinkage values are slightly different in the three axes. In particular, the values found are in line with those found by other authors using analogous systems, as reported in Table 4. These values led to the revision of the *OFS* values (see Equation (1)) as follows: 1.26 for the plane and 1.30 for the perpendicular direction. Large amounts of organics (used as the base matrix in the production of filaments) necessitate lengthy thermal cycles for de-binding processes in parts produced by FFF, which causes significant shrinkage in sintered parts and has also been designated as a limitation for the fabrication of ceramics by AM [30,31,40,41]. For these reasons, it is important to produce filaments with precise dimensional tolerances, manage over-extrusion, and assess how the presence of ceramic powder and moisture affect the susceptibility of organic components to degradation.

### 3.3. Mechanical Properties and Fracture Analysis of Sintered Samples

**Figure 6:**
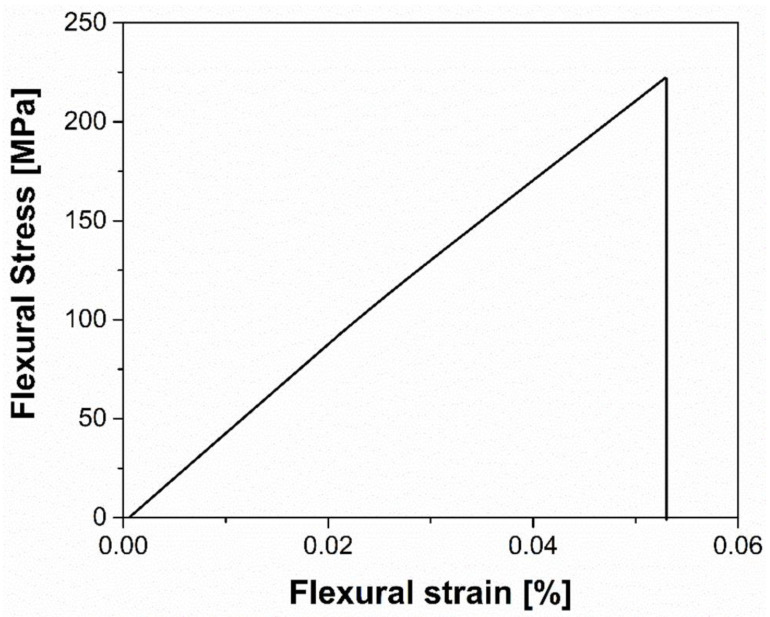
Representative flexural stress–strain curve of Al_2_O_3_ 3D-printed sintered sample.

All the sintered samples were tested under flexural load, and the results were compared to analogous techniques. Table 4 reports the comparison with other properties, such as density, average shrinkage, and hardness. Figure 6 reports a representative flexural curve for the 3D-printed sintered alumina sample.

The material shows a brittle behaviour typical of ceramics, with no plastic deformation being recorded. The mean values of flexural strength, the strain at rupture, and the elastic modulus (E) computed from the stress–strain curve are reported in Table 5.

Alumina bulk ceramics typically have flexural strengths between 450 and 550 MPa. As a result, according to the standard four-point bending test, alumina falls into the category of intermediate ceramics, between ZnO (100 MPa) and Si3N4 (900 MPa) [44]. It should be noted that the flexural strength of ceramics, and hence of alumina, is highly dependent on the manufacturing process. For instance, pressure-free sintering of high- purity alumina TM DAR produced pure alumina with a relative density of less than 99 percent, reaching 520 MPa [45]; robocasting was used to achieve a flexural strength of only 156.6 MPa [70], while Michalek et al. [60] achieved value of 856 MPa using CIM and hot press. Moving on to the AM printed alumina, the mechanical properties change considerably. In fact, with respect to FFF printing, Orlovskà et al. [47] printed three-point bending samples with layer heights of 100 and 300 microns, finding flexural strengths of 300 and 200 MPa, respectively. With the same material used in this study, Truxovà et al. [51] obtained a flexural strength of 331.61 MPa, while a range of 200–500 MPa was claimed by the manufacturer [50]. Considering other AM techniques (i.e., stereolithography or binder jetting), values of flexural strength by four-point bending equal to 427 and 367.9 MPa for Schwentenwein et al. [48] (LCM) and Dehurtevent et al. [58] (Stereolithography—SLA—process), respectively, have been reported. Considering the mechanics of ceramic, the presence of porosity both of nano and micro size highly influences the origination and growth of cracks, leading to premature failure [71]. As shown by the SEM investigation, there are some porosities in our samples as a consequence of the FFF process. These porosities act as defects, lowering mechanical resistance. Cano et al. [72] investigated how the infill orientation affected the characteristics of AM ceramic parts made using FFF. Inter-filament holes, defects caused by material over extrusion, and nozzle shearing in the deposited material have all been identified as common FFF shaping flaws that are the primary determinant for variations in values of density (95–98%), porosity (2.5–5.3%) and bending strength (366–512 MPa). The measured Vickers hardness of the 3D-printed sintered Al_2_O_3_ was 21.6 GPa. This value is in agreement with those obtained by other researchers [50,51], but slightly lower than bulk alumina obtained via traditional manufacturing, showing HV of 25 GPa [73]. The sintering temperature is the main parameter influencing the mechanical properties; in our case, the relatively low sintering temperature of 1550 °C justifies the obtained hardness value [74].

**Figure 7:**
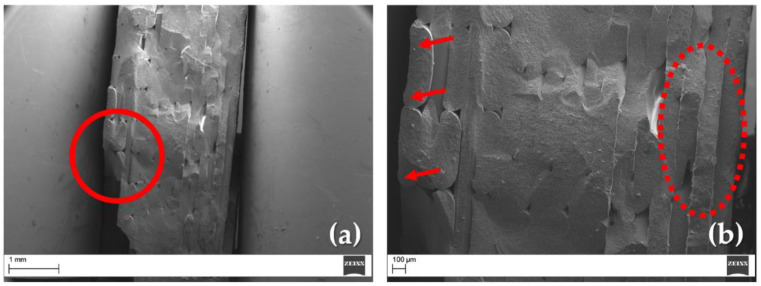
SEM analysis of the sintered sample’s fracture surface: (**a**) initial crack point (red circle); (**b**) FFF-induced defects (red arrows) and bottom layers (red dashed ellipse).

One way to reduce porosity in FFF printing is to increase the amount of extruded material, a technique called overextrusion [75,76]. However, as noted by Cano et al. [77], this choice can cause a “flooding” of the material between rasters, as shown by the arrows in Figure 7b. The appearance of these types of defects [76,77], more pronounced in the top layers than in the bottom layers (indicated by red dashed ellipse in Figure 7b), leads to the onset of preferred fracture start points, as indicated by the red circle in Figure 7a. Therefore, the defects induced by the FFF printing process play an essential role compared to the intrinsic ones of the material. Greater attention to the multiple FFF parameters appears to be important in order to reduce defects, increase the density of the printed parts, and thus increase the thermo-mechanical properties of the final sintered parts.

### 3.4. Thermal Conductivity Properties of Sintered Samples

The thermal diffusivity test was carried out on three sintered samples, and the thermal conductivity was calculated as described in Section 2.3. In Table 6, the mean and standard deviation values are reported for all the temperatures investigated in the range 25–350 °C.

The thermal conductivity values shown in the Table 6 fall within the range 7.82–21.52 [W/(mK)]. These values are below those reported for traditional techniques, i.e., 33 [61] or 32 [78] [W/(mK)] at room temperature. Taking into account the porosity of the parts, Honda et al. [79] reports thermal conductivity values equal to 34.3, 25, 16.4 and 11.8 [W/(mK)] for porosities equal to 3, 12, 25, 40%, respectively. Similarly, as reported by Azo Materials [80] the measured thermal conductivity values were equal to 28–35, 26, 24, 20 and 15 [W/(mK)] for densities equal to 99.9, 99.5, 97.5, 94 and 86%, respectively.

As a result, it is possible that the presence of flaws and porosity in the green and sintered samples contributed to slightly reducing heat transfer within the material.

In fact, due to the internal pores and air gaps visible in Figure 5c,d, FFF printed parts are intrinsically anisotropic, even at 100% infill. These imperfections therefore restrict layer-to-layer contact and, as discussed in a previous paper [55], often cause undesirable thermal property degradation.

The thermal conductivity analysis proved to be a useful test for investigating the quality of the printed parts in terms of the presence of voids. Further investigations must be carried out to correlate the printing and process parameters with the thermal properties of the green and sintered parts.

### 3.5. Comparison with Other AM Techniques

With regard to the mechanical properties, the highest values achieved were those for conventional manufacturing. However, the advantage of AM production is evident, especially in the absence of dedicated equipment for each geometry to be created. Due to AM processes’ ability to manufacture advanced ceramic components without the use of moulds, significant cost savings are possible, especially for low-volume production.

Therefore, the results for the manufacture of alumina using FFF obtained in this study were compared with a vat photopolymerization AM process. Vat photopolymerization technologies, particularly SLA and DLP, have a high level of industrial readiness as their first advantage. In fact, the most well-established method for creating monolithic advanced ceramic components using additive manufacturing is currently light-activated polymerization. Lithoz [81], Prodways [82], 3DCeram and Admatec [83] are the principal industrial producers of machines based on this methodology. However, due to excessive light scattering and light absorption, which causes poor resolution and insufficient polymer curing, ceramic materials with even higher refractive indexes and higher coefficients of extinction have proven to be more difficult to shape [62]. The maximum wall thickness that can be produced using photopolymerisation-based processes may also be constrained in terms of the absence of porosity or cracks. In fact, the photocurable ceramic slurries used in SL and DLP share a lot in common with the suspension feedstock used in injection moulding. They typically contain at least 40 to 60 percent by volume of polymeric binder as the suspension medium, to which other organic substances like dispersants and photoinitiators are added. Due to the release of gaseous species that must gradually escape, the majority by means of diffusion, these large amounts of organics have a tendency to complicate the debinding stage, in addition to first causing very significant shrinkage, which may result in deformation of the part. As a result, processing thick-walled parts in the brown body without excessive porosity and cracking is difficult [84]. Additionally, using a lot of toxic photopolymers has negative effects on health and safety and the environment.

**Figure 8:**
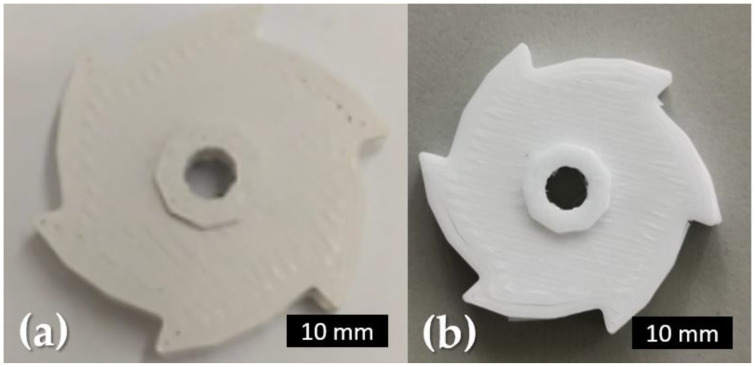
FFF alumina: (**a**) green part; (**b**) sintered part.

For the cost-assessment analysis, a cost model from Prof. Jonathan Hart of Massachusetts Institute of Technology (MIT) was used [85]. It is necessary to consider the costs and/or time spent on the following: equipment, materials, build preparation, production, labour, and postprocessing. Purchase, operating, and maintenance fees from the equipment manufacturer are all included in the machine costs. Feedstock costs are included in the cost of materials, while build preparation and post-processing costs largely depend on how complex the additive manufacturing process is. It is challenging to make accurate calculations for the D&S processes, and this difficulty increases when considering ceramic additive manufacturing. For these reasons, the comparison only considered the printing of green parts. A production size of 100 alumina parts (Figure 8) was assumed, produced using four printing techniques (Table 7).

For the four printing techniques, LCD, DLP, LCM and FFF, the 3D printers Precision Ceramic 1.5 (research version), Tethon 3D Bison 1000^TM^ and Raise 3D Pro2 Plus, respectively, were considered. The first factor to consider is the print volume capacity; in fact, for printers using the LCD, DLP, LCM and FFF techniques, the build volumes were 1.2, 0.9, 0.6 and 23.8 L, respectively. This first value provides an indication of the production capacity; in fact, the number of builds required to satisfy the order of 100 parts for the LCD, DLP, LCM and FFF techniques was 20, 25, 50 and 2, respectively. Therefore, it is necessary to dedicate only two print jobs to completing the prints by FFF printing. In addition to the productivity value, the print volume gives us an idea of the freedom of the geometries to be printed, keeping in mind the dimensional constraints currently present for the D&S process. Another parameter of definite importance is the cost of the machine and, therefore, its usage. The most expensive machine is the Lithoz Cerafab 7500 [77,86], followed by the Bison 1000 [87], the Precision Ceramic 1.5, and the Raise3D Pro2 Plus [88]. This explains the high value of machine usage (Table 7) with respect to the LCM technique. Considering other values related to the printing technique, such as the amount of support needed, which is almost always a factor for VAT Photopolymerization techniques, but is avoidable when using the FFF technique, the Total cost can be obtained. The Total cost divided by the number of parts gives us the Average Cost per Part (ACP) value, which is useful for comparing printing techniques from an economic point of view. The cheapest technique is FFF, with an ACP of just EUR 9.71. Conversely, the most expensive techniques are DLP, LCM and LCD, with ACP values equal to EUR 46.61, EUR 81.16 and EUR 94.62, respectively. These differences can be explained, in addition to the Machine cost, by the price of the printing materials. For the LCD technique, in fact, this price is the highest, as it is a product still under development, purchased by the research group and investigated in a previous work [89]. These price differences flatten out when considering the DLP and LCM techniques. Therefore, the DLP technique can certainly be considered a more expensive alternative (more than 4 times) to FFF, in this case taking into consideration the printer Bison 1000^TM^ by Tethon 3D, operating with Bison High-Alumina Resin.

## 4. Conclusions

The tool-free AM technique under study is a solid and effective method for making prototypes or small quantities of ceramic objects.

Functional AM ceramics still have limitations in terms of their density; an alumina crystalline phase with a mean density of 3.80 g/cm^3^ can be observed in the sintered samples. Although significant research efforts have been made to produce dense alumina, widespread application of this knowledge in industrial settings still requires further experimental work and significant financial investment. The thermo-mechanical properties are mainly affected by FFF printing and the D&S parameters.

The sintered ceramic parts exhibited a tensile strength of 232.6 ± 12.3 MPa and a Vickers hardness of 21 ± 0.7 GPa. The mean value of thermal conductivity at room temperature was equal to 21.52 ± 0.02 W/(mK). The values obtained through FFF production were lower than those obtained through conventional processes despite the sintering cycle, because the mesostructured of the 3D-printed samples exhibited the voids and imperfect interlayer bonding common to FFF printing.

To maximize density and predict and limit shrinkages, the authors advise using filaments with tight dimensional tolerances, managing the flow ratio, and assessing the impact of ceramic powder content and moisture on the degradation of the organic components. As a result of the lengthy thermal cycles required for the de-binding processes due to the high levels of organics in the FFF parts, significant shrinkage in the sintered parts was also identified as a limitation for the fabrication of ceramics using AM technology.

Furthermore, as demonstrated by the authors on the basis of a cost assessment of a real FFF printed and sintered part, the recently developed highly filled ceramic filament is suitable for use in cost-effective and simple-to-manage FFF machines. Photopolymerization techniques (SLA, DLP, LCD, LCM) are widely regarded to be state-of-the-art technologies for ceramic additive manufacturing, but their high costs, lengthy processing times, and limited material selection currently prevent them from being used more widely in industry. Prior to VAT processes, FFF has become a well-established and affordable AM technology. However, FFF is constrained by inherent process limitations and has relatively low resolution and poor surface finish. Since it is based on modelling thermoplastic feedstocks, it can take advantage of the information already accumulated by using conventional injection moulding techniques. As a result, FFF provides a more affordable method for producing ceramic components, enabling the manufacture of multi-materials with no limitations on material choice and build volume.

Furthermore, an open road in research concerns the development of 3D-structured functional ceramics, suitable for a variety of applications in the fields of catalysis and energy systems, through the functionalization of ceramic-based feedstock materials with secondary additives.

## Figures and Tables

**Table 2:** Debinding process parameters.

Segment	Starting Temperature (°C)	Ending Temperature (°C)	Heating Rate (°C/h)
1	20	125	35
2	125	200	50
3	200	215	22
4	215	250	11
5	250	280	20
6	280	320	8
7	320	510	24

**Table 3:** Dimensions of green and sintered parts, with actual shrinkages. Mean values are reported, with standard deviations in brackets.

Axis	Green Part Length *L_g_* [mm]	Sintered Part Length *L_s_* [mm]	Shrinkage [%]
x	10.035 (0.04)	7.961 (0.05)	20.6
y	10.085 (0.01)	7.972 (0.03)	20.9
z	10.175 (0.08)	7.843 (0.09)	22.9

**Table 4:** Properties of alumina 3D-printed parts using different technologies compared to those in this study.

References	Production Method	Density	Average Shrinkage	Flexural Strength	Hardness (HV10)
	-	%Th	%	MPa	GPa
Current study	FFF	95–98	20.7 (xy), 22.9 (z)	232.6 ± 12.3	21.6 ± 0.7
Nötzel et al., 2020 [52]	FFF	97	20.75 ± 0.8	-	-
Orlovskà et al. [47]	FFF	86–89	19	200 (0.3 mm)–300 (0.1 mm)	-
Nötzel et al., 2019 [54]	FFF	99.4 (50 vol%) 99.6 (55 vol%)	22.8 ± 1.2 (45 vol%) 20.4 ± 1.4 (50 vol%), 18.0 ± 1.6 (55 vol%)	-	-
Gorjan et al. [53]	FFF	>98.73	23 (z) 17 (plane)	144–147 (disc test, not 4PB)	-
Noetzel et al., 2018 [19]	FFF	97.3	-	-	-
Schwentenwein et al. [48]	VAT (LCM)	99.3	19.68	427 (4PB)	14.22
Dehurtevent et al. [58]	VAT (SLA)	98.1	14 ± 1.8 (z), 19.1 ± 0.7 (y), 17.2 ± 0.3 (x); overall 16.9 ± 2.3	367.9 (4PB)	-
Rueschhoff et al. [69]	Robocasting	98 (55 vol%) 98.2 (56 vol%)	<0.2%	133.6 ± 17.8 (56 vol%); 156.6 ± 17.5 (55 vol%)	-
Rowthu et al. [59]	Slip casting method	87–99.5	14 (z) 11 (xy)	262–561 (4PB)	-
Michàlek et al. [60]	CIM + Hot pressed	>99	-	856 (4PB)	-
Munro et al. [70]	Sintered	>98	-	380	15
Truxová et al. [51]	FFF	99.54	22.5	331.61 (100% infill)	23.81 (100% infill)
Nanoe [50]	FFF	98–99	20.8 ± 1 (xy), 23.2 ± 1 (z)	200–500	19

**Table 5:** Mechanical properties of 3D-printed sintered alumina.

Flexural Strength [MPa]	Strain at Break [%]	Vickers Micro Hardness [HV]	Micro Hardness [GPa]
232.6 ± 12.3	0.055 ± 0.003	2206 ± 71	21.6 ± 0.7

**Table 6:** Mean values of thermal conductivity. Standard deviations are reported in brackets.

Temperature [°C]	Thermal Conductivity [W/(m K)]
25	21.52 (0.02)
50	18.25 (0.01)
100	12.84 (0.01)
150	11.07 (0.03)
200	10.86 (0.01)
250	9.44 (0.02)
300	8.45 (0.03)
350	7.82 (0.02)

**Table 7:** Cost model of LCD, DLP, LCM, and FFF printing of alumina.

Voice of Cost	LCD (Photocentric Precision Ceramic 1.5)		DLP (Tethon 3D Bison 1000^TM^)		LCM (Lithoz Cerafab 750)		FFF (Raise 3D Pro2 Plus)	
Material	80.5%	EUR 7614	54.1%	EUR 2523	42.9%	EUR 3485	20.6%	EUR 200
Build preparation	2.2%	EUR 210	4.5%	EUR 210	2.6%	EUR 210	7.2%	EUR 70
Machine usage	0.1%	EUR 10	0.8%	EUR 36	14.0%	EUR 1136	0.6%	EUR 6
Build consumables	0.3%	EUR 30	0.8%	EUR 38	0.9%	EUR 75	0.1%	EUR 1
Labor	11.0%	EUR 1043	27.9%	EUR 1299	32.7%	EUR 2656	14.3%	EUR 139
Post-process	5.9%	EUR555	11.9%	EUR 555	6.8%	EUR 555	57.1%	EUR 555
Total cost		EUR 9462		EUR 4661		EUR 8116		EUR 971
Average cost per part		EUR 94.62		EUR 46.61		EUR 81.16		EUR9.71

## Data Availability

Not applicable.
